# Deep amplicon sequencing of preselected isolates of *Parascaris equorum* in β-tubulin codons associated with benzimidazole resistance in other nematodes

**DOI:** 10.1186/1756-3305-7-410

**Published:** 2014-08-29

**Authors:** Eva Tydén, Johan Dahlberg, Olof Karlberg, Johan Höglund

**Affiliations:** Department of Biomedical Sciences and Veterinary Public Health, Division of Parasitology, Swedish University of Agricultural Sciences, Uppsala, S-750 07 Sweden; Department of Medical Sciences, Molecular Medicine and Science for Life Laboratory, Uppsala University, Uppsala, S-751 44 Sweden

**Keywords:** *Parascaris equorum*, Anthelmintic resistance, β-tubulin, SNP, Fenbendazole, Illumina HiSeq

## Abstract

**Background:**

The development of anthelmintic resistance (AR) to macrocyclic lactones in the equine roundworm *Parascaris equorum* has resulted in benzimidazoles now being the most widely used substance to control *Parascaris* infections. However, over-reliance on one drug class is a risk factor for the development of AR. Consequently, benzimidazole resistance is widespread in several veterinary parasites, where it is associated with single nucleotide polymorphisms (SNPs) in drug targets encoded by the β-tubulin genes. The importance of these SNPs varies between different parasitic nematodes, but it has been hypothesised that they occur, at low allele frequencies, even in unselected populations. This study investigated whether these SNPs exist in the *P. equorum* population and tested the hypothesis that BZ resistance can develop from pre-existing SNPs in codons 167, 198 and 200 of the β-tubulin isotype 1 and 2 genes, reported to be associated with AR in strongylids. The efficacy of the oral paste formula fenbendazole on 11 farms in Sweden was also assessed.

**Methods:**

Two isotype-specific primer pairs were designed, one on either side of the codon 167 and one on either side of codons 198 and 200. A pool of 100 000 larvae was sequenced using deep amplicon sequencing by Illumina HiSeq. Faecal egg count reduction test was used to assess the efficacy of fenbendazole.

**Results:**

No SNPs were observed in codons 167, 198 or 200 of the β-tubulin isotype 1 or 2 genes of *P. equorum*, even though 100 000 larvae were sequenced. Faecal egg count reduction testing of fenbendazole showed that this anthelmintic was still 100% effective, meaning that the likelihood of finding high allele frequency of SNPs associated with benzimidazoles resistance in *P. equorum* was low. Unexpectedly, the allele frequencies observed in single worms were comparable to those in pooled samples.

**Conclusions:**

We concluded that fenbendazole does not exert selection pressure on the β-tubulin genes of isotypes 1 and 2 in *P. equorum*. The fact that no pre-existing SNPs were found in codons 167, 198 and 200 in *P. equorum* also illustrates the difficulties in generalising about AR mechanisms between different taxonomic groups of nematodes.

**Electronic supplementary material:**

The online version of this article (doi:10.1186/1756-3305-7-410) contains supplementary material, which is available to authorized users.

## Background

*Parascaris equorum* is a nematode parasite of foals and yearlings with a global distribution. It has a direct life cycle and is one of the most important equine parasites because of its high prevalence and severe pathogenicity [1]. For many years, this ascarid roundworm could be controlled by strategic use of different anthelmintics, including macrocyclic lactones (ML), benzimidazoles (BZ) and tetrahydropyrimidines (TH) [2]. However, since the ML ivermectin (IVM) was first introduced in the beginning of the 1980s many horses have been dewormed with this compound up to six times during their first year, which has resulted in the development of ML resistance in the *P. equorum* population [3]. Furthermore, reduced efficacy of the TH pyrantel pamoate (PYR) in ML-resistant *P. equorum* isolates has been reported in the U.S. [4, 5]. In contrast, the BZs fenbendazole (FBZ) and oxibendazole (OBZ) are still effective and is the anthelmintic drug class currently recommended for treatment of *P. equorum* infection [6].

It has been shown repeatedly that overuse of any anthelmintic can lead to selection of resistance-associated alleles, as experienced for different intestinal nematode parasites of livestock and horses [1]. For BZ, it is generally accepted that certain single nucleotide mutations (SNP) induce changes in amino acids, which may lead to structural changes in the drug target [1, 3]. For many nematodes of veterinary interest, in particular *Haemonchus contortus* of sheep, BZ resistance has been associated with SNPs in the β-tubulin isotype 1 molecule, leading to reduced binding of BZ and thereby loss of anthelmintic efficacy [7]. In *H. contortus*, three different SNPs have been reported to be responsible for BZ resistance: substitution of phenylalanine (TTC) by a tyrosine (TAC) at (1) codon 200 [8–11] or at (2) codon 167, also leading to a shift from TTC by TAC at codon 200 [12], or (3) a change in glutamate (GAA) to alanine (GCA) at codon 198 [13]. Homozygous resistance mutations at more than one locus have never been found, which suggests that single mutations in the β-tubulin gene are tolerated, whereas two or more mutations make the protein dysfunctional and are lethal for the parasite [14].

The mechanisms of AR development in nematodes essentially remain unknown, but it is understood that AR can arise in three different ways [15, 16]. Mutations in drug targets may appear spontaneously and can then spread within the parasite populations through gene flow, for example together with trade of livestock [17]. Thus mutations occur randomly and at a rate, which depends on the reproduction rate and the population size of the organism [7]. Whether the mutations are then maintained in the population depends on balancing selection [15]. For example, pre-existing polymorphisms in the parasite population could be equally selected by unilateral drug treatment, resulting in a rapid increase in the frequency of resistant alleles. The origin of BZ resistance in equine nematodes is generally unknown, but the presence of a TAC mutation in codon 167 has been reported in cyathostomins, even in those from horses that have never received any anthelmintic treatment [18]. Whether this indicates that TAC polymorphism was pre-existing or occurred as a result of gene flow from another population exposed to BZ is unclear [18]. Similar data on the role of β-tubulin and the development of BZ resistance in other equine nematodes such as *P. equorum* are lacking.

There are currently no studies reporting BZ resistance in *P. equorum*, but over-reliance on one drug class (in this case BZ) is regarded as a risk factor for AR development [1]. There is only one anthelmintic (fenbendazole) currently available which is still effective against *P. equorum* in Sweden. Therefore drug rotation is not possible, leading to an increased risk of selection of BZ resistance. The first objective of the present study was thus to test the hypothesis that BZ resistance in *P. equorum* can develop from pre-existing SNPs in codons 167, 198 and 200. This was investigated using the Illumina HiSeq next generation sequencing (NGS) technique on a pool of 100 000 eggs from both pre-selected and unselected isolates of the parasite obtained from Sweden and the U.S. The second objective was to examine the anthelmintic efficacy of oral paste formula fenbendazole (FBZ) on farms in Sweden, as currently recommended for treatment of *P. equorum* in foals (http://www.sva.se/).

## Methods

**Figure 1:**
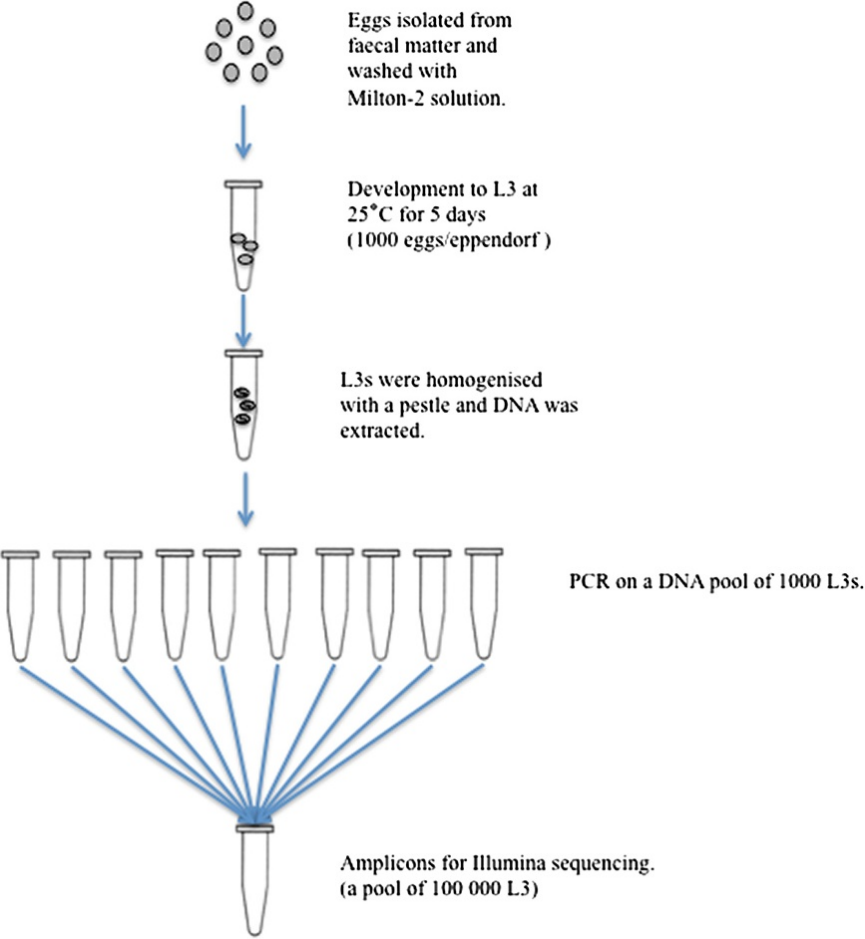
**An overview of the experimental design for the pool of 100**,**000 L3.**

For an overview of the experimental design, see Figure 1.

### Parasite material

Eggs were collected in October-December from the 11 Swedish farms included in the FECRT. Most foals were about 6–8 months and had been treated with both IVM and FBZ according to the deworming recommendations issued by the Swedish Veterinary Institute. Furthermore, the U.S. isolate was obtained from a closed research horse herd maintained at the University of Kentucky without anthelmintic intervention since 1979 [18], and the parasites maintained there are therefore anthelmintic-naïve. The second U.S. isolate (U2) was collected during routine necropsy at the Veterinary Diagnostic Laboratory, University of Kentucky. The worms from the U.S. were preserved in ethanol (70-90%) and sent by airmail to the laboratory at the Division of Parasitology, Swedish University of Agricultural Sciences, Uppsala. Upon arrival, all worms were stored at -70°C until DNA extraction was performed.

### Isolation and washing of eggs from faecal material

*Parascaris* eggs were isolated from foal faecal matter by sieving with different mesh sizes (1000 μm, 150 μm) and finally collected in an 80 μm sieve. The eggs were further cleaned by centrifugal flotation and then diluted in tap water and stored in ventilated cell culture flasks at 7°C.

### DNA extraction

Prior to DNA extraction, the eggs were washed with Milton-2 (sodium hypochlorite 2% (v/v) in sodium chloride 16.5%) solution at 1500 rpm for 3 minutes and thereafter washed with cold water several times. To increase the DNA yield, the eggs were incubated at 25°C for 5 days to develop into third stage larvae (L3). Eggs developed into L3 were divided into 1000 eggs/eppendorf tube and homogenised in 0.2 mL buffer using a plastic pestle. Complete crushing of the eggs was confirmed by light microscopy. DNA was extracted using Nucleospin Tissue (Macherey-Nagel, Düren, Germany) according to the manufacturer´s instructions. The DNA extraction process described above was also performed on each individual U.S. worm. The purified DNA was stored at 4°C until further analysis.

### Primer design

**Figure 2:**
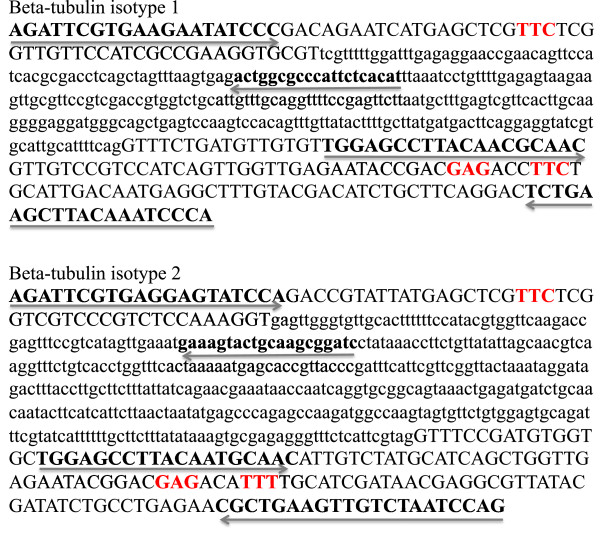
**Position of the primers**, **indicated by arrows and bold font.** The codons 167, 198 and 200 are marked in red. Capital letters indicate the exons and lowercase letters indicate the sequenced intron between the two exons.

The Illumina TruSeq DNA library preparation kit used for preparation of sequencing libraries requires an amplicon size of minimum 120 bp. To achieve the right size of amplicon, the intron between the exon containing the 167 codon and the exon containing the 198 and 200 codons was cloned and sequenced using the Big Dye® Terminator v3.1 Cycle Sequencing Kit Protocol (Applied Biosystems), according to the manufacturer’s instructions. The sequences were determined using a Genetic Analyser (ABI PRISM® 3100). The intron sequences for β-tubulin 1 and β-tubulin 2 were edited and analysed with CLC Main Workbench version 5.6.1. A isotype-specific primer pair was designed on either side of the codon 167 and another isotype-specific primer pair on either side of codons 198 and 200 according to Figure 2.

### PCR

To enable even amplification from all individuals in the pool, a touchdown PCR was carried out under the following conditions: 25 μL reaction volumes containing 5 μL of the DNA pool, 10 mM Tris–HCl pH 8.3, 4 μg Bovine Serum Albumin (BSA), 50 mM KCl, 2 mM MgCl_2_, 0.4 μM of each primer, 0.24 mM dNTP, and 1.5 U AmpliTaq GOLD DNA polymerase. All reagents were from Applied Biosystems apart from BSA, which was supplied by New England Biolabs. The touchdown cycling parameters for the amplification consisted of an initial denaturation at 95°C for 5 min, followed by 10 cycles of 95°C for 30 sec, primer annealing at 60–50°C for 30 sec with an decrease of 1°C/cycle, and extension at 72°C for 1 min, followed by another 20 cycles for 95°C for 30 sec, primer annealing at 50°C and extension at 72°C for 1 min, with a prolongation of the final extension of 5 min (Bio-Rad, My Cycler^TM^ thermal cycler). After PCR, amplicons (a pool of 1000) were analysed by electrophoresis on Tris-Borate electrophoresis buffer (TBE) 1.5% agarose gels. Negative water controls were included in all runs. Later, the amplicons were pooled to contain 100,000 individuals and thereafter purified using Qiaquick (Qiagen). PCR amplification of the pool of 30 adult worms was performed as described above. As a check of the methodology, PCR amplification for one individual adult worm was run in parallel. Prior to Illumina sequencing, the 100,000 samples were quantified on Victor (Wallac 1420 VICTOR^2^_TM_, software version 2.0) using PicoGreen ds DNA quantification kit (Molecular Probes) and diluted to 14 ng/μL in a total volume of 50 μL.

### Amplicon library preparation for Illumina

Illumina HiSeq compatible sequencing libraries were prepared using the TruSeq DNA Sample Preparation Kit v2 (Illumina) according to the manufacturer’s instructions, with the exception that the PCR products were used directly without a fragmentation step and no size selection was done before library amplification. The sequencing libraries were constructed with individual barcodes and all libraries were pooled and sequenced in a single lane on an Illumina HiSeq2000.

### Data analysis

Each amplicon was sequenced to a minimum of 480 Mbp over Q30, yielding a coverage of at least of one million X per amplicon. The raw reads were aligned to their corresponding amplicon using bwa 0.6.2 (bwa aln with -q5 and bwa sampe with standard parameters) [19]. A custom tool to extract the codons of interest from each read was developed on top of the GATK Framework [20]. It was required that every base in the codon should be of Q30 quality or higher, otherwise the whole read was excluded from the analysis. Codon frequency was then determined as the fraction of the codon compared with the total number of codons observed.

### Faecal egg count reduction test

A total of 110 foals, 10 foals/farm, bred and housed on 11 large stud farms in Sweden, were included in the faecal count reduction test (FECRT). The study took place in autumn 2011, after weaning. The mean age of the foals was 6.5 months, and they had been dewormed 2 or 3 times during the summer prior to the study. In the test, the foals were treated with FBZ as an oral paste at the standard dose rate of 7.5 mg/kg body weight (Axilur®vet., Intervet Boxmeer, Netherlands). The weight of the foals was estimated visually by an experienced horse manager and rounded up to the nearest 50 kg. Faecal samples were collected from each foal on the day of treatment (day 0) and again 14 days post-treatment (day 14), placed in plastic bags and sent to the laboratory at the Division of Parasitology, Swedish University of Agricultural Sciences, Uppsala. Samples were stored overnight at 8°C before analysis. The number of ascarid eggs per gram faeces (EPG) was determined both before and after treatment by a modified McMaster technique, using saturated salt as the flotation medium (specific gravity 1.2) and with lowest detection level 50 EPG [21]. Calculations of the efficacy of the FBZ were performed with the FECRT according to the formula: 1-(mean EPG_day0_/mean EPG_day14_) × 100%. Reductions were calculated using the arithmetic mean for each farm. Since there is no internationally agreed standard for evaluating the FECRT for *P. equorum*, an isolate was considered resistant when the observed reduction was ≤90% and the lower confidence limit was ≤90%, and if only one criterion was met AR was suspected. This is in accordance with thresholds used for other equine nematode parasites and suggestions by [1].

## Results

### β-tubulin allele frequencies in codons 167, 198 and 200

The allele frequencies in a *Parascaris* L3 larval pool, a U.S. pool and individual worms were compared. No SNPs associated with BZ resistance were observed in codons 167, 198 or 200 in the genes of β-tubulin isotype 1 (Table 1) or isotype 2 (Table 2). A similar low allele frequency as reported in the L3 larval pool and the U.S. pool was also observed in the individual worms analysed here (Additional file 1: Table S1). As such a low variation should not be present in individual worms it was most likely the result of context-dependent PCR artefacts.

**Table 1:** **Allele frequency** (%) **in codons 167**, **198 and 200 of isotype 1**

	Isotype 1		
	Codon 167/TTC	Codon 198/GAG	Codon 200/TTC
**Individual worm**	98.2	96.3	96.1
**L3 pool**	98.2	96.0	95.8
**U.S. pool**	98.2	96.1	95.8

**Table 2:** **Allele frequency** (%) **in codons 167**, **198 and 200 of isotype 2**

	Isotype 2		
	Codon 167/TTC	Codon 198/GAG	Codon 200/TTT
**Individual worm**	98.0	98.1	97.3
**L3 pool**	98.3	98.1	97.4
**U.S. pool**	98.1	98.1	97.3

A consistent difference was observed in the genetic code of codon 200 between isotypes 1 and 2, with isotype 1 having TTC and isotype 2 having TTT. This difference had no impact on resistance, because TTC and TTT both code for the amino acid phenylalanine.

### FECRT

Mean efficacy of the oral paste formula FBZ in reducing *P. equorum* EPG was tested in foals on 11 stud farms in Sweden. The FECRT showed 100% efficacy in reducing EPG on all farms (Table 3).

**Table 3:** **Results of Faecal Egg Count Reduction** (**FECR**) **test evaluating the efficacy of fenbendazole paste** (**7.5 mg**/**kg**) **on 11 stud farms in Sweden**

Geographical location	Group mean EPG ^*^ day 0	Group mean EPG day 14	Max EPG day 0	FECR ^**^
**Boden**	3070	0	7450	100%
**Borlänge**	140	0	450	100%
**Heby**	1580	0	3250	100%
**Bro**	55	0	200	100%
**Ekerö**	950	0	2500	100%
**Tystberga**	100	0	550	100%
**Västervik**	275	0	500	100%
**Västervik**	950	0	1250	100%
**Landskrona**	250	0	550	100%
**Svenljunga**	1070	0	2200	100%
**Sjöbo**	970	0	1150	100%

Most foals had a previous history of two treatments of FBZ and IVM prior to collection of *Parascaris equorum* eggs.

*EPG, eggs per gram faeces, **FECR = (1-(EPG_d14_/EPG_d0_)) × 100%.

## Discussion

With the emergence of ML resistance, FBZ (a BZ) is the recommended drug for treatment of *P. equorum* infection in foals in Sweden. The results from the FECRT of FBZ on Swedish farms showed that no eggs appeared in the faeces 14 days post-treatment and this anthelmintic was still 100% effective. There are no previous reports of BZ resistance in *P. equorum*, possibly because the use of BZ has been restricted for therapeutic applications due to the widespread BZ resistance in cyathostomins since the mid-1970s [2]. However, owing to the altered conditions today with widespread ML resistance, over-reliance on BZ must be considered an important risk factor that can lead to the emergence of multiple AR in *P. equorum*.

Based on SNPs in codon 200 in other nematodes of veterinary interest, BZ resistance is likely to be a recessive trait [9]. Therefore in light of the results from the FECRT, there was little likelihood of finding high allele frequencies of SNPs associated with BZ resistance, particularly not in the unselected pool of *P. equorum*. A total of 100,000 eggs developed to L3 stage were sequenced, but no variation was observed in codons 167, 198 or 200 in β-tubulin isotypes 1 or 2. Although the origin of BZ resistance in nematodes is unknown, it has been hypothesised that resistant alleles already exist within the parasite population, even prior to drug selection [10]. Furthermore, in cyathostomins the presence of TAC mutation in codon 167 is reported to occur in parasites recovered from horses that have never received anthelmintic treatment [22]. Similar findings have been made for the whipworm *Trichuris trichiura*, with the presence of TAC mutation in codon 200 having been identified in the population of Haiti prior to albendazole (ABZ) treatment, and in an ABZ-naïve *T. trichiura* isolate from humans in Kenya [23, 24]. No such pre-existing genetic variation in codons 167, 198 or 200 was observed in the *P. equorum* isolates studied here. Phylogenetic analysis of currently known isotypes has shown that representatives of the Nematoda exhibit more/greater diversity among the β-tubulin genes than representatives of the Vertebrata [25]. Based on that observation, it can be stated that associations of genetic data with BZ resistance cannot be generalised from one taxonomic group to another. This statement is further supported by the results obtained in the present study. Overall, the lack of BZ-associated SNPs in the *P. equorum* population observed in this study, but also in the related ascarid *Ascaris lumbricoides* [23], *Trichurus* spp. [26] and several strongylids such as *Trichostrongylus tenuis* [27], *Necator americanus*, *Ancylostoma duodenale* and *A. caninum* [28], confirms the need for caution when generalising about BZ resistance between different nematodes.

Recent studies in *H. contortus* have revealed associations between the selection for ML resistance and the BZ-associated SNPs in the β-tubulin gene [29]. This suggests that ML resistance could appear as a result of selection for BZ resistance [29]. However, although the *Parascaris* pool screened for SNPs in this study was collected from several farms with documented ML resistance [30, 31], there was no evidence of a similar mechanism in *Parascaris*. In contrast to *H. contortus*, it appears that ML resistance does not select for the BZ-associated SNPs in the β-tubulin genes of *P. equorum*. This further confirms that generalisations cannot be made regarding the mechanisms for selection of resistance between different species of nematodes.

A most important finding was the difference between the nucleotides in codon 200 in isotype 1 (TTC) and isotype 2 (TTT). This codon can thus be used to distinguish isotype 1 from isotype 2, but it has no other impact because both TTC and TTT code for the same amino acid, phenylalanine.

Next generation sequencing (NGS) is a novel technique for mass screening of SNPs in pooled samples, since it permits rapid, deep sequencing of hundreds to thousands of DNA samples. This range of analysis is essential for uncovering the full spectrum of genetic variants occurring at low allele frequencies [32]. Accordingly, NGS is envisioned to be applied in various clinical applications, where quantitative screening of SNPs is important [33]. To date, routine molecular diagnostic testing for BZ resistance in nematode parasites of veterinary importance has mainly been based on pyrosequencing [22, 34]. NGS has multiple advantages over conventional cloning or the single genome sequencing techniques used for semi-quantification of rare genetic variants because of its faster speed and greater depth of coverage [35]. Based on the results obtained for individual worms sequenced by NGS in parallel to the pools, we were able to conclude that errors introduced by PCR artefacts were more important for the detection limit than the current sequencing accuracy. Overall, the results from this study show that deep sequencing of amplicons with NGS provides the power to detect rare alleles when screening a large sample pool.

## Conclusions

Reliance on one drug class poses a threat of multiple AR developing in *P. equorum*, especially in light of recent findings on the genetic homogeneity of the global *Parascaris* population and the high gene flow within this population [36]. In the present study, a candidate drive approach was used to screen for SNPs known to confer BZ resistance. Despite the increased use of BZ for treatment of *Parascaris* infection, SNPs in the β-tubulin genes known to confer resistance in other nematodes were clearly absent in *P. equorum*. In studies seeking new potential markers for resistance, a non-candidate driven approach would be an option for determining how AR arise. New potential markers for AR could hopefully be identified for the taxonomic group Ascaridoidea by the use of genome-wide expression profiles before and after exposure of *P. equorum* to anthelmintics.

## Electronic supplementary material

Additional file 1: Table S1: Allele frequency in codons 167, 198 and 200 of isotope 1 and 2. (XLS 134 KB)

## Abbreviations

ABZ: Albendazole
AR: Anthelmintic resistance
BZ: Benzimidazoles
EPG: Eggs per gram faeces
FECRT: Faecal egg count reduction testing
FBZ: Fenbendazole
ML: Macrocyclic lactones
NGS: Next generation sequencing
OBZ: Oxibendazole
SNPs: Single nucleotide polymorphisms.
